# The International Scientific Association of Probiotics and Prebiotics (ISAPP) consensus statement on the definition and scope of postbiotics

**DOI:** 10.1038/s41575-021-00440-6

**Published:** 2021-05-04

**Authors:** Seppo Salminen, Maria Carmen Collado, Akihito Endo, Colin Hill, Sarah Lebeer, Eamonn M. M. Quigley, Mary Ellen Sanders, Raanan Shamir, Jonathan R. Swann, Hania Szajewska, Gabriel Vinderola

**Affiliations:** 1 Functional Foods Forum, Faculty of Medicine, University of Turku, Turku, Finland; 2 Institute of Agrochemistry and Food Technology-National Research Council (IATA-CSIC), Valencia, Spain; 3 Department of Food, Aroma and Cosmetic Chemistry, Faculty of Bioindustry, Tokyo University of Agriculture, Hokkaido, Japan; 4 School of Microbiology, University College Cork, Cork, Ireland; 5 APC Microbiome Ireland, University College Cork, Cork, Ireland; 6 Department of Bioscience Engineering, University of Antwerp, Antwerp, Belgium; 7 Division of Gastroenterology and Hepatology, Lynda K and David M Underwood Center for Digestive Disorders, Houston Methodist Hospital and Weill Cornell Medical College, Houston, TX USA; 8 International Scientific Association for Probiotics and Prebiotics, Centennial, CO USA; 9 Institute of Pediatric Gastroenterology, Nutrition and Liver Diseases, Schneider Children’s Medical Center, Petach Tikva, Israel; 10 Sackler Faculty of Medicine, Tel Aviv University, Tel Aviv, Israel; 11 School of Human Development and Health, Faculty of Medicine, University of Southampton, Southampton, UK; 12 Department of Metabolism, Digestion and Reproduction, Imperial College London, London, UK; 13 Department of Paediatrics, The Medical University of Warsaw, Warsaw, Poland; 14 Instituto de Lactología Industrial (CONICET-UNL), Faculty of Chemical Engineering, National University of Litoral, Santa Fe, Argentina

**Keywords:** Microbiota, Microbiome

## Abstract

In 2019, the International Scientific Association for Probiotics and Prebiotics (ISAPP) convened a panel of experts specializing in nutrition, microbial physiology, gastroenterology, paediatrics, food science and microbiology to review the definition and scope of postbiotics. The term ‘postbiotics’ is increasingly found in the scientific literature and on commercial products, yet is inconsistently used and lacks a clear definition. The purpose of this panel was to consider the scientific, commercial and regulatory parameters encompassing this emerging term, propose a useful definition and thereby establish a foundation for future developments. The panel defined a postbiotic as a “preparation of inanimate microorganisms and/or their components that confers a health benefit on the host”. Effective postbiotics must contain inactivated microbial cells or cell components, with or without metabolites, that contribute to observed health benefits. The panel also discussed existing evidence of health-promoting effects of postbiotics, potential mechanisms of action, levels of evidence required to meet the stated definition, safety and implications for stakeholders. The panel determined that a definition of postbiotics is useful so that scientists, clinical triallists, industry, regulators and consumers have common ground for future activity in this area. A generally accepted definition will hopefully lead to regulatory clarity and promote innovation and the development of new postbiotic products.

## Abstract (web summary)

Postbiotics are emerging substances prepared from inactivated microorganisms, in contrast to probiotics, which must be administered alive. This Consensus Statement outlines a definition for the term ‘postbiotics’ as determined by an expert panel convened by the International Scientific Association for Probiotics and Prebiotics.

## Introduction

The past few decades have demonstrated unequivocally the importance of the human microbiota to both short-term and long-term human health. Early programming of the microbiota and immune system during pregnancy, delivery, breastfeeding and weaning is important and determines adult immune function, microbiome and overall health^1^. We have also seen rapid growth in the number of products that claim to affect the functions and composition of the microbiota at different body sites to benefit human health.

Improving human health through modulation of microbial interactions during all phases of life is an evolving concept that is increasingly important for consumers, food manufacturers, health-care professionals and regulators. Microbiota-modulating dietary interventions include many fermented foods and fibre-rich dietary regimens, as well as probiotics, prebiotics and synbiotics, some of which are available as drugs and medical devices, as well as foods^2^. The rich, diverse microbial ecosystems and immune cells inhabiting all mucosal and cutaneous surfaces provide targets for intervention, with the goals of reducing the risk of diseases and improving health status^2^. Consensus definitions of probiotics, prebiotics and synbiotics have been published previously. Probiotics are “live microorganisms that, when administered in adequate amounts, confer a health benefit on the host”^3^, whereas a prebiotic is a “substrate that is selectively utilized by host microorganisms conferring a health benefit”^4^. A synbiotic, initially conceived as a combination of both probiotics and prebiotics, has now been defined as “a mixture comprising live microorganisms and substrate(s) selectively utilized by host microorganisms that confers a health benefit on the host”^5^. The concept of postbiotics is related to this family of terms and is emerging as an important microorganism-derived tool to promote health.

Probiotics are by definition alive and required to have an efficacious amount of viable bacteria at the time of administration to the host, but most probiotic preparations, especially at the end of shelf life, will also include potentially large numbers of dead and injured microorganisms^6,7^. The potential influence of non-viable bacterial cells and their components on probiotic functionality has had little attention.

Fermented foods might also contain a substantial number of non-viable microbial cells, particularly after prolonged storage or after processing, such as pasteurization (for example, soy sauce) or baking (for example, sourdough bread). Food fermentation has a major influence on the physical properties and potential health effects of many foods, especially milk and plant-based foods^8^. Many fermentations are mediated by lactic acid bacteria, which can produce a range of cellular structures and metabolites that have been associated with human health, including various cell surface components, lactic acid, short-chain fatty acids (SCFAs) and bioactive peptides among other metabolites^9^. These effector molecules of fermented food microorganisms are thought to be similar to those produced by probiotics, but this link has not been conclusively established. In parallel, bacterial lysates of common bacterial respiratory pathogens have been used for decades to prevent paediatric respiratory diseases by postulated general immune-stimulating mechanisms that are not yet well understood^10^. The possibility that non-viable microorganisms, their components and their end-products play a part in the health benefits of such products is the rationale underlying the need for accurate terminology. We consider that a common understanding of the emerging concept of postbiotics, including a consensus definition, would benefit all stakeholders and facilitate developments of this field. Herein, we address several aspects pertaining to postbiotics, including processing factors important in their creation, proper characterization, mechanistic rationale on how they work to improve both intestinal and systemic health, safety and current regulatory frameworks. Key conclusions from this consensus panel are provided in Box 1.

Box 1 Main conclusions of the consensus panel regarding postbiotics

- A postbiotic is defined as a “preparation of inanimate microorganisms and/or their components that confers a health benefit on the host”.
- Postbiotics are deliberately inactivated microbial cells with or without metabolites or cell components that contribute to demonstrated health benefits.
- Purified microbial metabolites and vaccines are not postbiotics.
- A postbiotic does not have to be derived from a probiotic for the inactivated version to be accepted as a postbiotic.
- The beneficial effects of a postbiotic on health must be confirmed in the target host (species and subpopulation).
- The host can include humans, companion animals, livestock and other targets.
- The site of action for postbiotics is not limited to the gut. Postbiotics must be administered at a host surface, such as the oral cavity, gut, skin, urogenital tract or nasopharynx. Injections are outside the scope of postbiotics.
- Implicit in the definition of a postbiotic is the requirement that the postbiotic is safe for the intended use.

## Methods

ISAPP, a non-profit collaboration of scientists dedicated to advancing the science of probiotics and prebiotics, convened an expert panel of basic and clinical scientists to address the emerging concept of postbiotics in December 2019. ISAPP activities are determined by a volunteer academic board that functions independently of industry supporters of the organization. The panel comprised experts in probiotics and postbiotics, adult and paediatric gastroenterology, paediatrics, metabolomics, regulatory affairs, microbiology, functional genomics, cellular physiology of probiotics and host interactions and/or immunology. Prior to the meeting, panellists agreed on the relevant questions. During the meeting, panellists presented perspectives and evidence, debated the proposed questions and reached consensus. After the meeting, individual panellists wrote sections of this paper and the major contributions were as follows: S.S., regulatory aspects and background; H.S., paediatric health, nutrition and systematic reviews; R.S., paediatrics and evidence-based recommendations; A.E., Japanese and Asian history of postbiotics; C.H., microbiology and mechanisms; M.C.C., food microbiology and human milk postbiotics; S.L., mechanisms of postbiotic action and comparison with other substances; J.R.S., mechanisms and metabolomics; G.V., technological aspects of postbiotic measurement; E.M.M.Q., preclinical and adult evidence; and M.E.S., implications for stakeholders and regulatory considerations. These sections were discussed and modified by all panellists together and finally compiled by S.S. and M.E.S. into a draft report. This document was edited and agreed upon by all panel members, and finally by the non-author members of the ISAPP Board of Directors, D. Merenstein, R. Hutkins, K. Scott, G. Gibson and M. Marco.

## Proposed definition of postbiotic

**Fig. 1:**
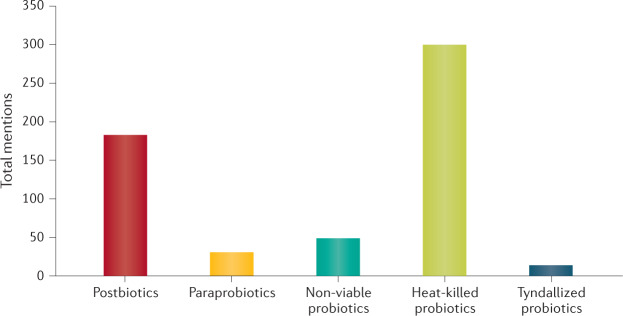
Total number of mentions in the literature of different terms referring to inanimate microorganisms and/or their metabolites. Several different terms, all defined differently, have been used over the years to refer to some form of inactivated or killed microorganisms in the research literature according to a search of the literature found on PubMed for the period 1 January 2000 to 21 January 2021. Bacterial lysates were not included in the search although they may be considered postbiotics if health benefits are documented and other criteria for postbiotics are met; the isolation of lysates is also a procedure in molecular biology studies that is often used in situations unrelated to postbiotics, so the term could not be used unambiguously in this search. The data that support the plots within Fig. 1 are available from the authors upon reasonable request.

**Fig. 2:**
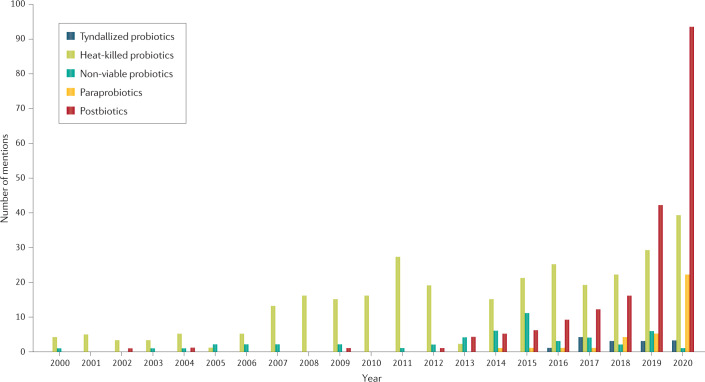
Increasing use of the term postbiotics in the published literature. Several different terms have been used over the years to refer to some form of inactivated or killed microorganisms in the research literature according to a search of the literature found on PubMed for the period 1 January 2000 to 21 January 2021. During the past 5 years, ‘postbiotics’ has emerged as the most common of these terms. The data that support the plots within Fig. 2 are available from the authors upon reasonable request.

The term postbiotic was chosen by the panel as a composite of ‘biotic’, defined as “relating to or resulting from living organisms”, and ‘post’, a prefix meaning ‘after’. Together these terms suggest ‘after life’; that is, non-living organisms. The concept that non-living microorganisms could promote or preserve health is not new, and several terms have been used to describe such substances, although during the past decade, postbiotic has been used most often (Figs 1,2). Other related terms have also been used, including ‘paraprobiotics’^11–14^, ‘parapsychobiotics’^15^, ‘ghost probiotics’^16^, ‘metabiotics’^17,18^, ‘tyndallized probiotics’^19,20^ and ‘bacterial lysates’^21^. However, the field would benefit from coalescing around the use of a single, well-defined and understood term rather than the use of disparate terms for similar concepts. We suggest that the term ‘postbiotic’ be used when applicable.

We propose that a postbiotic is a “preparation of inanimate microorganisms and/or their components that confers a health benefit on the host”. Alternative definitions of this word have been proposed (Table 1), but we believe this consensus definition best fits the understanding of this concept. This wording was chosen following substantial debate and consensus building. We chose to use ‘inanimate’, meaning lifeless, rather than ‘inactive’ as this latter term might suggest an inert material. ‘Inanimate’ simply captures the fact that live microorganisms were present but have now been killed, without implying a loss of function. ‘Preparations’ was chosen to reflect the likelihood that a specific formulation of microbial biomass, the matrices and/or inactivation methods have a role in any beneficial effects. The term ‘postbiotic’ would, therefore, be reserved for specific preparations, which would include descriptions of the microorganisms, the matrix and the inactivation method that had collectively contributed to a demonstrated health benefit. The word ‘components’ was included because intact microorganisms might not be required for health effects, and any effects might be mediated by microbial cell components, including pili, cell wall components or other structures. The presence of microbial metabolites or end products of growth on the specified matrix produced during growth and/or fermentation is also anticipated in some postbiotic preparations, although the definition would not include substantially purified metabolites in the absence of cellular biomass. Such purified molecules should instead be named using existing, clear chemical nomenclature, for example, butyric acid or lactic acid. Vaccines, substantially purified components and products (for example, proteins, peptides, exopolysaccharides, SCFAs, filtrates without cell components and chemically synthesized compounds), and biological entities such as viruses (including bacteriophages) would not qualify as postbiotics in their own right, although some might be present in postbiotic preparations. To qualify as a postbiotic, the microbial composition prior to inactivation must be characterized, and so preparations derived from undefined microorganisms are not included in the definition. For example, many traditional fermented foods are made through the action of undefined, mixed cultures, and such a product could not be used for the preparation of a postbiotic. However, postbiotics could be derived from fermented products made using defined microorganisms. The criteria for a preparation to qualify as a postbiotic are shown in Box 2.

**Table 1:** Past proposed definitions of the term ‘postbiotics’

Definition	Microbial cells included	Ref.
Any factor resulting from the metabolic activity of a probiotic or any released molecule capable of conferring beneficial effects to the host in a direct or indirect way	No	^176^
Soluble factors (products or metabolic byproducts), secreted by live bacteria, or released after bacterial lysis, such as enzymes, peptides, teichoic acids, peptidoglycan-derived muropeptides, polysaccharides, cell surface proteins and organic acids	No	^177^
Compounds produced by microorganisms, released from food components or microbial constituents, including non-viable cells that, when administered in adequate amounts, promote health and well-being	Yes	^178^
Non-viable metabolites produced by probiotics that exert biological effects on the hosts	No	^179^
Non-viable bacterial products or metabolic byproducts from probiotic microorganisms that have positive effects on the host or microbiota	Yes	^180^
Functional bioactive compounds, generated in a matrix during fermentation, which may be used to promote health	Yes	^181^

Many existing postbiotics include inanimate strains belonging to established probiotic taxa within some genera of the family Lactobacillaceae (now comprising 31 genera^22^) or the genus *Bifidobacterium*^23–25^. However, a microbial strain or consortium does not have to qualify as a probiotic (while living) for the inactivated version to be accepted as a postbiotic. Specific strains of *Akkermansia muciniphila*, *Faecalibacterium prausnitzii*, *Bacteroides xylanisolvens*, *Bacteroides uniformis*, *Eubacterium hallii*, *Clostridium* cluster IV and XIVa, *Apilactobacillus kunkeei* and the fungus *Saccharomyces boulardii* have all been investigated for potential beneficial effects in an inanimate form and would fit the definition of postbiotic should a health benefit be demonstrated^26–30^. Many bacterial lysates have been used for medical purposes, but there is a clear need for more robust clinical trials. For example, a report by the European Medicines Agency (EMA) describes the assessment of eight different lysates developed for respiratory conditions^31^. The report provides a review of the results of clinical studies, data on adverse effects reported with these medicines, and advice from an expert group on infectious diseases and considers the benefit–risk balance of bacterial lysate-based products. Based on this review, EMA recommended that bacterial lysate medicines authorized for respiratory conditions should only be used for the prevention of recurrent respiratory infections and not for treatment or pneumonia. The companies must also provide further data on safety and effectiveness from new clinical studies by 2026. A commercial oral postbiotic developed to protect against a variety of respiratory pathogens through boosting immune function illustrates the possible microbiological complexity of postbiotic design^32^. For this preparation, 21 different bacterial strains are grown in individual batches, heat-inactivated once they reach a critical mass, harvested, and then subjected to alkaline lysis and further purification steps^33^. The microbiological composition includes one strain of *Haemophilus influenzae*, four strains of *Streptococcus pneumoniae*, two strains of *Klebsiella pneumoniae* subsp. *pneumoniae*, one strain of *Klebsiella pneumoniae* subsp. *ozaenae*, two strains of *Staphylococcus aureus*, one strain of *Streptococcus pyogenes*, three strains of *Streptococcus sanguinis* and three strains of *Moraxella catarrhalis*. Bacterial lysates have further been shown to exert anti-infection effects^34^ and, indeed, efficacy in reducing the frequency of acute respiratory infections among those prone to recurrent respiratory infections has been demonstrated in clinical trials^34,35^. In addition, polyvalent bacterial lysates derived from the mechanical lysis of strains commonly involved in respiratory infections such as otitis media, pharyngitis, sinusitis and sometimes pneumonia induced the maturation of dendritic cells, recruit B and T lymphocytes, increase the number of circulating natural killer cells in treated patients when compared with age-matched controls^30^ and induced the secretion of specific IgA^36–38^ in a group of ten healthy volunteers, suggesting some potential in the treatment of chronic pulmonary conditions such as chronic obstructive pulmonary disease. Unfortunately, a large randomized placebo-controlled clinical trial with the related lysate in 288 patients (142 in the placebo group and 146 in the treatment group) failed to meet its primary end point — a reduction in exacerbations of chronic obstructive pulmonary disease^39^. Also, some spirulina formulations could qualify as postbiotics^40^, but only if the processing and microorganism used (often species *Arthrospira platensis*) is well described and the health benefit well documented in robust clinical trials.

Box 2 Criteria for a preparation to qualify as a postbiotic

- Molecular characterization of the progenitor microorganisms (for example, fully annotated genome sequence) to enable accurate identification and screen for potential genes of safety concern
- Detailed description of the inactivation procedure and the matrix
- Confirmation that inactivation has occurred
- Evidence of a health benefit in the host from a controlled, high-quality trial
- Detailed description of the composition of the postbiotic preparation
- Assessment of safety of the postbiotic preparation in the target host for the intended use

## Drivers of the postbiotic concept

### Stability

One important factor driving interest in postbiotics is their inherent stability, both during industrial processes and storage. Maintaining stability of live microorganisms is a technological challenge as many probiotic organisms are sensitive to oxygen and heat, but products with a long shelf life can be readily achieved for inanimate microorganisms. Postbiotics might also be more suited than probiotics to geographical regions that do not have reliable cold chains or whose ambient temperature causes problems for storage of live microorganisms.

For the majority of products with a long shelf life, probiotic die-off is inevitable during storage. Because the rate of death during storage depends on the physiological characteristics of the probiotic strain and the conditions of storage (time, temperature, water activity, oxygen levels, and others), it is difficult to generalize about the level of dead cells contained across probiotic products at the end of their shelf life. Responsible probiotic manufacturers often formulate their products with substantial overages to ensure that the labelled count of viable cells is met at the end of its shelf life. Even if such overages are not used, the live to dead ratio of a probiotic product can change substantially over the course of its shelf life^33^. Currently, probiotic product descriptions focus only on the viable cells in the product. This aspect raises some important questions. Is the efficacy of the product at the time of manufacture equivalent to the product at the end of the shelf life? What is the contribution of inanimate microorganisms to efficacy? These questions are especially important if the product is undergoing testing in a clinical evaluation. Although not common in the past, it seems important that going forward, quantifying the live and inactivated components of a probiotic product should be conducted over the course of an efficacy trial. Lastly, the safety of the probiotic must be assessed for the actual formulation amount, including overages. All of these concerns related to probiotic viability do not apply to postbiotics, which are likely be extremely stable for several years at room temperature and would be based on a fixed level of a viable microorganisms at the time of manufacture.

### Intellectual property protection

Another possible advantage of products devoid of live microorganisms is that the microorganisms from which the postbiotic is derived cannot be isolated from the commercial product, thereby enabling product developers to maintain ownership of their ingredients. However, the ability of researchers to reproduce findings is imperative for progress in this developing field and so we encourage researchers to make available the viable progenitor strains for research purposes, for instance, by depositing them in a public culture collection. The negligible level of viable microorganisms could also be an advantage in the development of postbiotics that might include genetically modified microorganisms, for which dissemination into the environment might be hazardous. Finally, if a postbiotic was derived from a microorganism from a country/region protected by the Nagoya Protocol (an international agreement that promotes sharing of benefits arising from biological resources in a fair and equitable way), the country of origin would be able to retain control of the microorganism.

### Regulatory considerations

To our knowledge, no regulators have advanced a postbiotic definition or framework specific to postbiotic-containing foods or food supplements. Some regulatory requirements have been advanced for postbiotic formulations whose intended use is directed towards medical or pharmaceutical applications^31^.

In Japan, postbiotics (termed ‘biogenics’ by Mitsuoka in 1998 (ref.^41^)) have been available for more than 100 years. Most of these products contain inanimate forms of lactic acid bacteria or bifidobacteria and are used in an assortment of food products, including juices, ice creams, popcorn, potato chips, natto (fermented soybeans), instant-type miso soup (traditional Japanese soup), supplements, tablets, pancake powder and many more. Most of these products are not associated with any health claims, but three products (two fermented-milk type drinks and a tablet) display health claims based on a regulation of Foods with Function Claims (FFC)^15,42–44^. The ingredient statements on such products might include, for example, lactobacilli, but they do not always state that the microorganisms added are non-viable. This type of labelling could mislead consumers concerning the content of the products.

Three regulatory approaches are possible for making health claims on foods in Japan: Food for Specified Health Uses (FOSHU), Foods with Nutrient Function Claims (FNFC) and FFC^45^. However, the FNFC is likely not applicable to postbiotics, leaving two possible routes . To date, no postbiotic food products have health claims based on FOSHU status but a few indicate health claims based on FFC are reported in the database of the Consumer Affairs Agency of Japan. Applications for FOSHU are reviewed and permitted by the Consumer Affairs Agency of the Government of Japan. Functional analyses and safety assessments of final products are essentially based on human studies. A permission seal from the authority appears on approved products. For FFC, scientific evidence is required from a systematic review of functional components or the product’s own clinical studies for applications. A history of the safe consumption of the species or scientific principles can be used to establish safety. A permission seal is not available for FFC^45^.

Postbiotics have had a long presence in Europe. Several postbiotics have been marketed or regulated as immune-stimulating agents^46^. However, in the European Union, no specific regulation covers probiotics, prebiotics, synbiotics or postbiotics. However, as we propose that their definition requires a health benefit, we expect that the use of any of these terms on a food or food supplement would require health claim approval. With regard to safety assessment in Europe, the European Food Safety Authority (EFSA) develops regularly updated lists of microorganisms that meet criteria for presumptive safety for use in foods. This process, called Qualitative Presumption of Safety (QPS), would apply to live microorganisms (including bacteria and yeast) used as progenitor microorganisms for postbiotics. Microorganisms not found on the list require a systematic novel food application and approval in Europe before they can be used for postbiotic development for foods or feeds. An example of a safety assessment of a potential postbiotic includes *B. xylanisolvens* for food^47^, which has undergone safety evaluations conducted on heat-treated or inactivated bacteria. For postbiotics formulated in medical products, the EMA (Directive 2004/27/EC)^48^) is in charge of both evaluation and supervision. For pharmaceutical preparations and medicinal products, the European Pharmacopoeia has clear criteria, which stipulate maximum allowed levels of live microorganisms^49^. Such criteria should be easily met by postbiotic products. The new EU Regulation 2017/745 (ref.^50^) for medical devices also has a specific paragraph positioning ‘living organisms’ out of the scope of the regulation but postbiotics do not seem to be out of scope.

In South America, Brazil has been the most active country in addressing probiotics and incorporating them in their regulations, publishing the first guidelines for their evaluation in 1999. Argentina did the same in 2011 and Chile in 2017 (ref.^51^). However, Brazil still takes the lead by updating their guidelines as they deem necessary according to the advancing knowledge on probiotics. The fact that Brazil was the first country/territory to address probiotic regulations, which have been updated several times over the past 20 years, could suggest that it might be the first in the region to incorporate postbiotics.

In Argentina, the Argentinian Food Code incorporated the concepts of probiotics and prebiotics in 2011 under Articles 1389 and 1390, respectively. However, the topic of postbiotics has not yet been addressed, even though in 2019 an international company launched an infant formula with 30% of its composition being derived from spray-dry-inactivated milk fermented with *Streptococcus thermophilus* and a *Bifidobacterium* strain, and the product was labelled ‘with postbiotics’. As in most cases, food development precedes regulation and, for regulation, a clear and well-accepted definition of postbiotics is needed.

In the USA, the Food and Drug Administration (FDA) has not specifically addressed postbiotics. A search shows no mention of the term ‘postbiotic’ on the FDA website. As postbiotics can be developed under different regulatory categories^52^, the FDA will probably approach postbiotics based on the regulations that pertain to the specific regulatory category chosen for a product under development. The product’s intended use, safety and efficacy will need to meet the standard for the applicable regulatory category. Thus, for example, if a postbiotic is to be used as a food ingredient, it will either need to undergo premarket approval as a food additive or need to be evaluated by experts to determine whether it is generally recognized as safe. Any health benefit claims made would need to be approved by the FDA either as a health claim, which identifies a food as able to reduce the risk of disease, or as a non-approved general function claim, which identifies a food as influencing the normal structure or function of the human body. Other regulatory categories that postbiotics could potentially fall under include drugs, medical devices or subcategories of foods, such as dietary supplements, infant formulas, foods for special dietary use or medical foods.

### Safety

Postbiotics could reasonably be expected to have a better safety profile than probiotics, because the microorganisms they contain have lost the capacity to replicate and therefore cannot cause bacteraemia or fungaemia, risks that are associated with probiotic administration (albeit extremely rare)^53^. However, postbiotics cannot be presumed to be safe solely based on the safety profile of the progenitor microorganism. For example, lipopolysaccharides from Gram-negative bacteria can induce sepsis and toxic shock, especially when endotoxin A, which is normally embedded in the outer membrane in living bacteria, is released from dead bacteria^54^. An assessment of safety for the intended use for any postbiotic is needed prior to use. Postbiotics derived from food-grade microorganisms or species in the continuously updated EFSA QPS lists might have an easier path to approval.

## Technological factors in characterization

Technological factors play an important part in how postbiotics are characterized and made. These factors include: accurate identification of the microorganisms used as the starting material for the postbiotic; description of the inactivation procedure or technique, as each process can result in a different postbiotic composition with different effects; and a description and quantification of the final postbiotic composition.

Postbiotics are inanimate by definition, and unless they are rapidly killed under the conditions used to make a product (for example, a strict anaerobe might not survive exposure to atmospheric conditions), they will require an inactivation step. A number of options are available to achieve this objective, and while this section lists some of the likely options, it is not an exhaustive list of available treatments that could inactivate microorganisms.

### Inactivation

Thermal processing is likely to be used in many instances to inactivate microorganisms, as there is a long history of thermal processing in the food industry. Traditional thermal processing (pasteurization, tyndallization, autoclaving) is widely used to confer enzymatic and microbiological stability on food systems. However, the temperature and length of time of heating affect nutritional value, sensory characteristics and flavour^55^. As a result, thermal processing might not always be optimal when generating a postbiotic preparation intended to be used as a food supplement or as a food.

Other processing technologies can provide useful alternatives to thermal sterilization or pasteurization^56^. Most of the technological knowledge concerning the non-thermal inactivation of microorganisms in foods was developed for the inactivation of food-borne microbial pathogens or spoilage microorganisms, but these technologies could be used equally well for the production of postbiotics. Non-thermal inactivation techniques were designed to obtain safe and stable foods with preserved overall quality and value while maintaining their sensory characteristics close to those of their fresh equivalents. In this context, technologies such as electric field, ultrasonication, high pressure, X-rays, ionizing radiation, high-voltage electrical discharge, pulsed light, magnetic field heating, moderate magnetic field^55^ and plasma technology^57^ could all potentially be applied to inactivate microorganisms and generate postbiotics.

Spray drying is a method of producing a dry powder from a liquid or slurry by rapidly drying with a hot gas. Spray drying has been proposed as a low-cost alternative to freeze drying to develop dehydrated but viable microbial cultures^58^, and could be used with higher inlet and/or outlet temperatures to achieve microbial inactivation. Spray-dried infant formulas fermented with lactic acid bacteria and bifidobacteria, but not containing substantial amounts of viable bacteria in the final product, are widely available in many countries^59^. They can therefore be labelled as including postbiotics if they are in agreement with our proposed definition and criteria.

Other drying techniques, such as vacuum and fluidized bed drying, have been shown to stress microorganisms and decrease their viability^60^ and could potentially be used under harsher operative conditions to completely inactivate cultures. Even more effective microbial inactivation might be achievable by the combined or successive application of these milder technologies, applied either independently or in tandem with other stresses, such as mild temperature^61^.

**Fig. 3:**
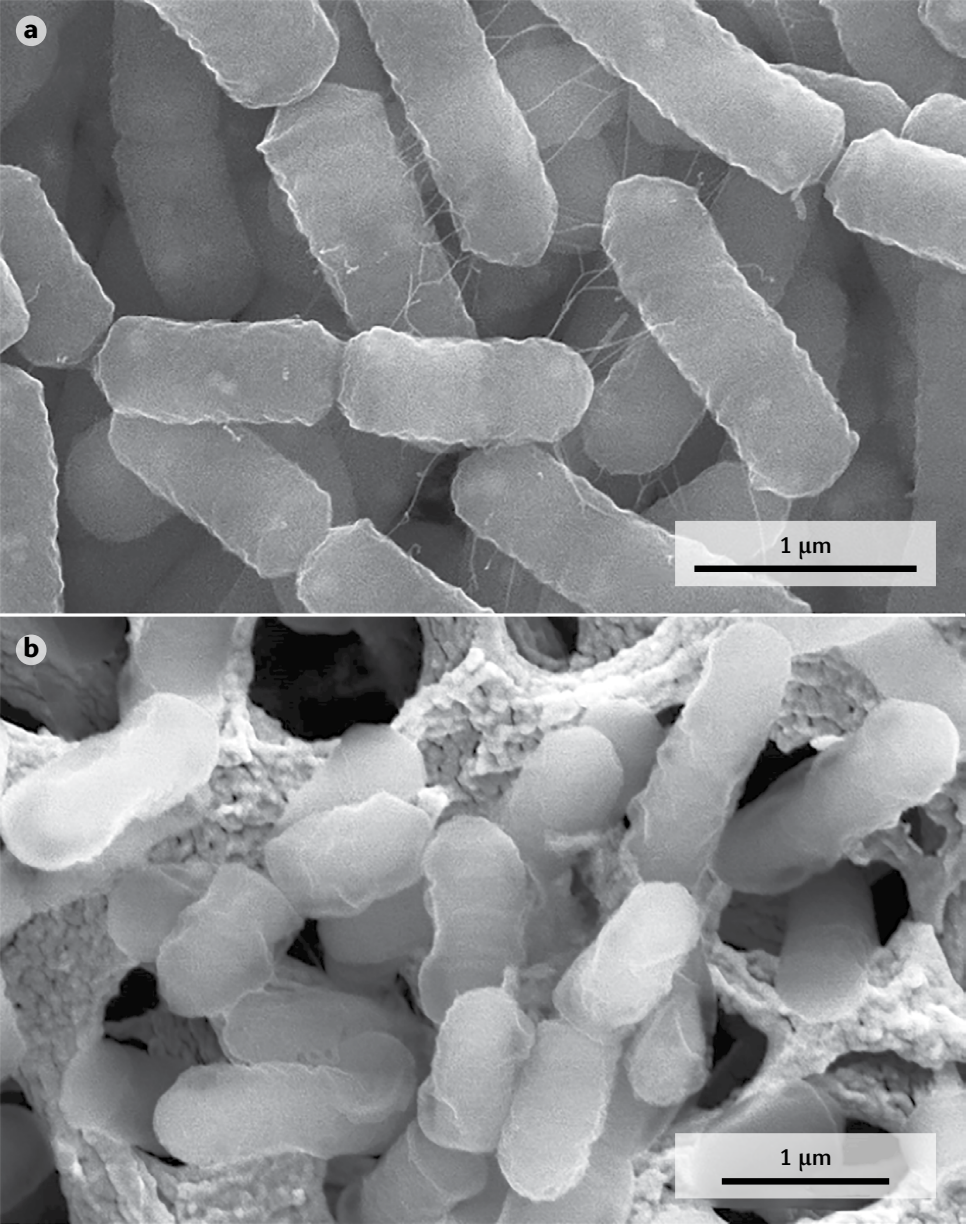
Scanning electron micrographs of *Lacticaseibacillus rhamnosus* GG. Scanning electron micrographs of *Lacticaseibacillus rhamnosus* GG in live (part **a**) and processed (part **b**) form showing that processing steps to obtain postbiotics can have a major effect on the physical and functional properties of the bacteria, even if the overall biomass and rod shape is preserved. Inactivation was performed in this case by spray drying that resulted in a mixture of live, full piliated cells and inactivated cells lacking pili surface appendages (as described in Kiekens et al.^75^). The bacteria were spotted on a gold-coated membrane, which is especially visible after processing. Adapted with permission from ref.^75^, Wiley.

In addition to the level of microbial inactivation achieved, the functionality of a postbiotic might be affected by the means of production. For instance, it has been shown that different heat treatments applied to the development of dehydrated probiotics (air drying, freeze drying and spray drying) can strongly affect both the viability and immunomodulatory properties of probiotic strains, and thus we can surmise that such treatments could also affect postbiotic properties^62^. Non-thermal treatments, such as high pressure, have also been reported to modify the in vivo host response to lactobacilli^63^. Figure 3 shows cells of *Lacticaseibacillus rhamnosus* GG (formerly known as *Lactobacillus rhamnosus*) before and after spray drying, which resulted in a mixture of live, fully piliated cells and inactivated cells lacking pili surface appendages. Pili are cell surface structures known to mediate bacterial–host immune interactions. For example, loss of pili has been linked to increased induction of pro-inflammatory markers such as IL-8 and less stimulation of cell proliferation and protection against radiologically inflicted intestinal injury in Caco-2 intestinal epithelial cells^64^.

We can learn much about the likely extent of microbial inactivation that can be achieved by thermal and non-thermal processing from studies conducted on food-borne pathogens. When heat is used, complete inactivation can be proportional to the level of heat and time of exposure, whereas in non-thermal food processing complete inactivation might not always occur in a linear fashion^65,66^. The extent of microbial inactivation depends on multiple factors related to the cell type (prokaryotes versus eukaryotes, Gram-positive versus Gram-negative bacteria, vegetative cells versus spores, cocci versus rod-shaped microorganisms), the processing conditions and the composition of the matrix^67^.

### Parameters for inactivation

Most postbiotics will contain no viable cells but some survivors might persist depending on the inactivation conditions^47^. Different inactivation technologies (heat, high pressure, exposure time to oxygen for strict anaerobic microorganisms) and procedures could be expected to result in different numbers of remaining viable cells of the progenitor microorganisms, although such comparisons have not yet been published. At the same time, extreme inactivation conditions designed to achieve complete inactivation might negatively influence the nutritional, physical, rheological or sensorial properties of the material. Thus, the inactivation method chosen could result in some residual, live microorganisms. Our intention is not to disqualify such products from our postbiotic definition. Although we do not require that a postbiotic be microbiologically sterile, there must be intentional and deliberate processing designed to inactivate the microbial progenitor strain. Here we do not suggest a precise limit on allowable live microorganisms remaining after postbiotic preparation as this is more appropriately a matter for regulators, as can be found in an EFSA assessment of *B. xylanisolvens*^47^.

### Quantification

Suitable methods must be available to describe the composition of and to quantify a postbiotic product. These methods must be available for clear product description to facilitate duplicative research as well as for quality control at the production site. Flow cytometry is emerging as an alternative to plate counting for microbial detection and enumeration^68^. In addition to being faster, it has the advantage of being able to separate a microbial population into live, damaged and dead cells. Results are expressed as total fluorescent units and active fluorescent units (AFUs). In flow cytometry, cells pass through a narrow aperture and they are analysed individually by a laser. A limitation of this counting method is that the correlation between AFUs and colony-forming units (CFUs) is not established, especially when applied to inactivation treatments that might produce several large fragments from a single cell (Fig. 3). Potentially, one cell rendering several fragments could be counted as several AFUs. In cases in which an AFU to CFU ratio of 1:1 is not expected owing to the disintegration of the microbial cell after an inactivation treatment has been applied, cell counts before inactivation might be a useful method to report the concentration of the postbiotic in the final product. Alternative analytical methods to analyse and quantify microbial biomass include proteomics and enzyme-linked immunosorbent assay-based approaches^69^, real-time PCR^70^, flow cytometry^68^, droplet digital PCR^71,72^, NMR^73^, atomic force spectroscopy^74^, scanning electron microscopy^75^ and Fourier-transform infrared spectroscopy^76^, but they are not yet commonly used by industry.

Freshly grown microbial cultures displaying high levels of viable cells can sometimes contain a higher number of non-viable cells, even in the absence of any inactivation step^77^. The level of inactive cells will depend on the conditions of the biomass production process, such as the growth phase at harvesting, medium composition or the pH profile throughout fermentation. Thus, because postbiotics will be derived from both active and inactive cells, CFU counts prior to inactivation might not prove an effective means of defining the cell biomass of a postbiotic product. Because CFUs before the inactivation process could underestimate the true biomass, flow cytometry might be a more suitable method.

It is also possible that intact inactivated cells could interact differently with the immune system when compared with their cell wall and cell membrane fragments, because of the different conformation and avidity of the immune-interaction molecules^6^. In this scenario, the type of technology used to inactivate cells (regardless of whether intact cells or cell fragments are generated) might result in products with different functionality compared with the progenitor microbial product. For this reason, it is important that each postbiotic preparation is consistently produced using the same technological process as the one used in the study in which a health benefit was demonstrated. If the process is altered, it is important to ensure the resulting product will produce the expected health effect.

## Biomolecules mediating health effects

**Fig. 4:**
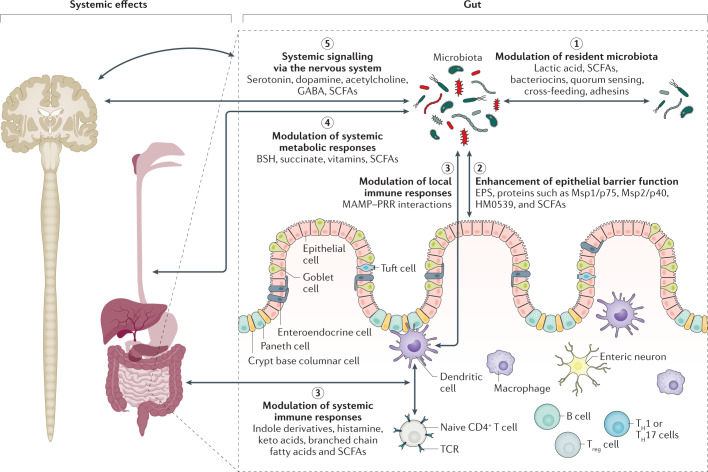
Postulated mechanisms of postbiotics and example effector molecules utilized by them. Five mechanisms of action of postbiotics are postulated: (1) modulation of the resident microbiota; (2) enhancement of epithelial barrier functions; (3) modulation of local and systemic immune responses; (4) modulation of systemic metabolic responses; and (5) systemic signalling via the nervous system. Some examples of microbial effector molecules mediating these mechanisms are shown (non-exhaustive list). Conceptually, the activity of effector molecules could be better retained if the cellular structure of the postbiotics is preserved, for example, through increased avidity in interactions with immune receptors or through increasing the residence time of the active molecules inside the host. The cell wall protects against rapid degradation by digestive enzymes and immune attack inside the host. This aspect is similar to the situation with vaccines, which also function best if cellular structure is preserved, but with the most toxic and/or pathogenic parts being inactivated or deleted. BSH, bile salt hydrolase; EPS, exopolysaccharide; MAMP, microbe-associated molecular pattern; PRR, pattern-recognition receptor; SCFAs, short-chain fatty acids; TCR, T cell receptor; T_H_ cell, T helper cell; T_reg_ cell, regulatory T cell.

The ability of a postbiotic, which can be a heterogeneous mixture of components, to mediate a health effect in the target host might be driven by many different mechanisms. In some cases, these mechanisms could be similar to those known for probiotics^3,78^. Such mechanisms might act independently or in combination. Understanding the major effector molecules involved in eliciting such beneficial effects is important information to ensure that a commercial postbiotic product retains the attributes necessary for efficacy. Because postbiotics are inanimate, these bioactive molecules must be synthesized by the progenitor microorganisms prior to inactivation, and in sufficient amounts to induce a beneficial effect. Here, we review possible mechanisms that could drive postbiotic efficacy. Overall, five main modes of action are considered, as depicted in Fig. 4.

### Beneficial modulation of microbiota

Although effects of postbiotics on the microbiota might be temporary, they could still have an important mechanistic role. Molecules present in postbiotics, such as lactic acid^79^ and bacteriocins^80^, can have direct antimicrobial activity according to in vivo studies. Postbiotics could also modulate the microbiota indirectly, for example by carrying quorum sensing and quorum quenching molecules^81^ or by carrying lactic acid that can be consumed by some members of the microbiota resulting in SCFAs and butyrate, which have a beneficial function^82^. Postbiotics can also compete with resident microorganisms for adhesion sites if the postbiotics provide adhesins (such as fimbriae^83^ and lectins^84^) that remain intact after processing.

### Enhancing epithelial barrier function

Activities that enhance epithelial barrier function can be mediated by secreted proteins, such as the major secreted proteins Msp1/p75 and Msp1/p40 (ref.^85^) or the protein HM0539 (ref.^86^) of the model probiotic *L. rhamnosus* GG. In addition, exopolysaccharides, such as those derived from *Bifidobacterium* species, can promote barrier function by reducing inflammation via yet-to-be defined signalling mechanisms^87^. Increasing evidence shows that certain *Bifidobacterium* species induce signalling pathways, such as MAPK and AKT, that promote tight junction functioning via autophagy and calcium signalling pathways^88^. SCFAs present in a postbiotic preparation have the potential to modify epithelial barrier function and protect against lipopolysaccharide-induced disruption, if present at sufficient levels^89^. For example, acetate (0.5 mM), propionate (0.01 mM) and butyrate (0.01 mM), alone or in combination, were shown to increase transepithelial resistance and stimulate the formation of tight junction in Caco-2 intestinal epithelial cells in vitro^89^. In another study, butyrate was demonstrated to alter the permeability of tight junctions via lipoxygenase activation through histone acetylation in Caco-2 cell lines^90^.

### Modulation of immune responses

Immune-modulatory activities at both local and systemic levels are generally exerted by microorganism-associated molecular patterns interacting with specific pattern recognition receptors of immune cells, such as Toll-like receptors (TLRs), nucleotide-binding oligomerization domain (NOD) receptors and C-type lectins, resulting in the expression of various cytokine and immune modulators^91^. The interactions of various microorganism-associated molecular patterns with specific immune receptors have been characterized, mainly via molecular interaction studies and validation in animal models: lipoteichoic acid interacting with TLR2 or TLR6 (ref.^92^); peptidoglycan or its derived muropeptides interacting with NOD2 (ref.^93^); fimbriae or pili modulating TLR2 signalling^64^; flagellae mostly interacting via TLR5 (ref.^94^); CpG–DNA interacting with TLR9 (ref.^95^); lipopolysaccharide of certain postbiotics derived from Gram-negative bacteria, such as *Escherichia coli* Nissle, mostly interacting with TLR4 and sometimes TLR2 (ref.^96^); β-glucans in yeast, such as *Saccharomyces cerevisiae*, interacting with TLR2 and lectin immune receptors^97^; and lipoproteins mostly interacting via TLR2 (ref.^98^). These microbe-associated molecular patterns could also be present in postbiotics if not destroyed or modified by the inactivation process. Some of the immunostimulatory bacterial lysate mixtures mentioned earlier contain lysates from both Gram-positive and Gram-negative bacteria and have been shown to interact with TLR4 and TLR2 (ref.^99^). In addition, metabolites, such as lactic acid, have been reported to mediate immune effects through, for example, the GPR31-dependent dendrite protrusion of intestinal CX3CR1^+^ cells^100^. Similarly, indole derivatives of tryptophan generated by *Limosilactobacillus reuteri* (formerly *Lactobacillus reuteri*) can activate the aryl-hydrocarbon receptor in CD4^+^ T cells in the mouse gut, inducing differentiation into CD4^+^CD8αα^+^ double-positive intraepithelial lymphocytes^101^. However, it is unknown whether indole derivatives are stably contained in postbiotic formulations. Other immunomodulatory microbial metabolites that could be present in postbiotics, based on molecular research in related microorganisms, include histamine^102^, branched chain fatty acids and SCFAs^103^, which have been shown to influence a number of immune responses, including suppression of NF-κB.

### Modulation of systemic metabolism

Effects on systemic metabolic responses can be directly mediated by the metabolites or enzymes inside and on the surface of the inactivated microorganisms in the postbiotics. One example is bile salt hydrolase (BSH). This microbial enzyme is responsible for the deconjugation of bile acids that enables further microbial biotransformation to occur, diversifying the overall circulating bile acid pool^104^. Bile acids can modulate the community structure of the microbiota generally and interact with various host receptors, with downstream effects on a range of host metabolic processes, including glucose, lipid, xenobiotic and energy metabolism^104^. BSH is predominantly expressed in the cytoplasm of microorganisms, but extracellular forms have also been observed, and its activity has been demonstrated in the filtered supernatant of the probiotic *Lactobacillus johnsonii*^105^. Interestingly, a loss of gut microbiota-derived BSH predisposes individuals to recurrent *Clostridioides difficile* infection, but restoration of this activity through faecal microbiota transplantation has been shown to assist in treating this infection, which was demonstrated in a study analysing stool samples from 26 patients and their 17 donors, followed by validation in a mouse model^106^. Another example is succinate, a bacterial intermediate of carbohydrate fermentation. Succinate is a substrate for intestinal gluconeogenesis that has been found to improve glycaemic control in mice^107^. Other known modulators of host metabolism include microbial-derived vitamins and SCFAs. Propionate can improve insulin sensitivity and glucose tolerance and modify lipid metabolism^108^, whereas butyrate can upregulate the antioxidant glutathione and can affect oxidative stress beneficially in the colon of healthy humans^109^.

### Signalling via the nervous system

Microorganisms can produce various neuroactive compounds that can act on both the enteric and central nervous systems with the potential to modulate behaviour and cognitive function in animals and humans^110^. This includes several neurotransmitters such as serotonin, dopamine, acetylcholine and GABA, and various compounds that can bind to receptors expressed in the brain (for example, indoles and bile acids). Microbial enzymes can also metabolize dietary precursors for host neurotransmitter synthesis (for example, tryptophan (for serotonin) and tyrosine (for dopamine)), reducing their bioavailability^111^. In addition, microbial metabolites, such as SCFAs, if present in a sufficient quantity in the postbiotic preparation, could stimulate enterochromaffin cells to produce serotonin, which can subsequently enter the bloodstream^112^. A study in mice and human enteroids using live and heat-killed *Bifidobacterium dentium* has highlighted that viability is crucial for serotonin induction by this microorganism^113^, so that it remains to be investigated whether postbiotic preparations other than heat-killed preparations could exert this effect. Moreover, SCFAs have been shown in human intervention studies to be able to modify feeding behaviours through the promotion of satiety by stimulating the release of anorexigenic hormones, such as glucagon-like peptide 1 and peptide YY^114,115^. In mice, gut-derived acetate has also been shown to enter the brain and regulate appetite through a central metabolic mechanism^116^. Bacterially synthesized vitamins, such as B vitamins (riboflavin, folate and cobalamin), can also be present in probiotics^117^ and probably also retained in postbiotics. B vitamins have important beneficial roles in central nervous system function^118^. However, how much of these neuroactive molecules are retained in postbiotics is not well documented at present.

We hypothesize that, as a general rule, the activity of effector molecules is increased if the cellular structure of the postbiotics is preserved, for example, through increased avidity in interactions with immune receptors or through increasing the residence time of the active molecules inside the host, because the cell wall protects against rapid degradation by digestive enzymes and immune attack inside the host, but further experimental proof is needed. This situation is similar to that with vaccines, which also function best if the cellular structure is preserved, but with the most toxic or pathogenic parts inactivated or deleted^119^. However, it cannot be ruled out that the activity and bio-availability of effector metabolites such as amino acid derivatives and SCFAs might be increased when the cellular structure is degraded owing to the molecules becoming more exposed and available.

## Health benefits of postbiotics

Postbiotics in general have been studied in the preventative and treatment contexts. Most of the research cited is in the medical field for therapeutic applications, but postbiotics could also have nutritional benefits. The following discussion focuses on preclinical studies and postbiotic-mediated benefits in adults and paediatric populations.

### Animal studies

The possibilities for postbiotics as clinical interventions have been well illustrated in the laboratory. Observations in animal models have, for some time, demonstrated biological activity of inanimate bacteria, which offer considerable formulation, safety and regulatory advantages over their ‘live’ counterparts. An example is a postbiotic derived from *Limosilactobacillus fermentum* and *Lactobacillus delbrueckii* that influenced behaviour in a mouse model. The fermentate was subjected to a high-temperature treatment to achieve microbial inactivation^120^. The postbiotic-fed animals demonstrated increased sociability and lower baseline corticosterone levels (stress hormone) and had subtle but statistically significant changes in the composition of their gut microbiota when compared with controls receiving a standard rodent chow. The study found that less abundant taxa were most affected. The same research group went on to use the same postbiotic in a mouse model of *Citrobacter*-induced colitis, which is characterized by a shortening of the small intestine and an increase in colon crypt depth^121^. The postbiotic did not prevent *Citrobacter* infection, but postbiotic-fed mice had a longer small intestine and reduced colon crypt depth compared with control animals that received standard mouse chow alone.

### Postbiotics in adults

#### Available evidence

For evidence on the health benefits of postbiotics in adults, the Cochrane Central Register of Controlled Trials and MEDLINE databases were searched for randomized controlled trials (RCTs), cohort studies, or their meta-analyses that compared postbiotics with placebos or no therapy. Data from human studies are limited but efficacy for orally administered, inactivated lactic acid bacteria has been demonstrated in the eradication of *Helicobacter pylori* infection^122^, reduction of symptoms in patients with irritable bowel syndrome (IBS)^25,123^ and chronic unexplained diarrhoea^124^, and in the abrogation of the negative effects of stress^15,125^. In a randomized, double-blind, placebo-controlled trial in 443 individuals with IBS involving orally administered, heat-inactivated *Bifidobacterium bifidum* MIMBb75, the postbiotic substantially alleviated symptoms associated with IBS, such as abdominal pain or discomfort, abdominal bloating and abnormal bowel habits^25^.

No benefits were seen in terms of modulating gut barrier function in 25 patients with increased permeability secondary to obstructive jaundice treated with inactivated *Lactiplantibacillus plantarum* (formerly known as *Lactobacillus plantarum*)^126^. Other inactivated strains, such as *Bacillus coagulans* (effect on responses to vigorous exercise among soldiers undergoing self-defence training)^127^, *Mycobacterium manresensis* (in tuberculosis)^128^, *Mycobacterium phlei* (in asthma)^129^ and *H. influenzae* (in severe chronic obstructive pulmonary disease)^130^ have also been studied in humans. As is the case with this entire category, data from human studies are limited, are of variable quality and have resulted in varying clinical impacts. *Mycobacterium vaccae* has attracted considerable attention because of the immunoregulatory and anti-inflammatory properties of the heat-killed microorganism, as demonstrated in the central nervous system, for example^131^. Others are also developing topical products with lysates of the probiotic *L. rhamnosus* GG for skin applications^132^. A preparation incorporating autologous platelet-rich plasma, biomimetic peptides, postbiotics (plantaricin A, *A. kunkeei* bee bread) and *Tropaeolum majus* flower, leaf or stem extract, was shown to be superior to placebo in the treatment of alopecia areata in 160 patients^133^. These preparations could therefore be termed skin postbiotics according to the new consensus definition. Further examples of postbiotics being used for therapeutic purposes in humans are delineated in Table 2.

**Table 2:** Examples of postbiotic use in adults

Country/region	Participants (*n*)	Intervention and control group	Duration of the intervention	Main conclusion	Ref.
***Inactivated bacteria***					
Italy	*Helicobacter pylori*-positive individuals (*n* = 120)	Triple therapy based on rabeprazole, clarithromycin and amoxicillin vs the same regimen supplemented with a lyophilized and inactivated culture of *L. acidophilus*	7 days	Eradication rates: triple therapy alone, 72%; triple therapy plus inactivated *L. acidophilus*, 87% (*P* = 0.02)	^122^
France	Patients with IBS with diarrhoea (*n* = 297)	Lacteol (inactivated *L. acidophilus* LB plus fermented culture medium), two capsules daily (no control)	1 month	Improved scores for pain, bloating, frequency of diarrhoea and quality of life	^123^
Germany	Patients with IBS (*n* = 443)	Non-viable, heat-inactivated *Bifidobacterium bifidum* MIMBb75 (SYN-HI-001) 1 × 10^9^ daily vs placebo	8 weeks	Composite primary end point of ≥30% improvement in pain and adequate relief of overall IBS symptoms in at least 4 of 8 weeks of treatment; primary end point achieved in 34% in active group vs 19% in the placebo group	^25^
China	Patients with chronic diarrhoea (*n* = 137)	Heat-killed *L. acidophilus* LB (Lacteol Fort), two capsules BID vs lacidophilin, five chewable tablets TID	4 weeks	Reduced stool frequency at weeks 2 and 4; overall symptoms improved at 4 weeks in Lacteol group	^124^
UK	Patients with obstructive jaundice (*n* = 25)	Oatmeal drink containing *Lactiplantibacillus plantarum* (formerly known as* Lactobacillus plantarum*) 299v (LP299v) vs oatmeal drink containing inactivated LP299v vs water	4 days	Measured intestinal permeability increased in water and inactivated groups; trend towards normalization in active group	^126^
Japan	Stress responses in undergraduate medical students taking a cadaver course (*n* = 32)	Heat-inactivated *L. gasseri* strain CP2305 in an acid beverage vs beverage alone	5 weeks	In male students, sleep quality was improved and diarrhoea prevented, but not in female students	^15^
Japan	Chronic stress responses in medical students (*n* = 60)	Heat-inactivated, washed and dried *L. gasseri* strain CP2305 (1 × 10^10^ bacterial cells per two tablets) vs placebo tablets once daily	24 weeks	Significant reduction (*P* < 0.05) in anxiety and sleep disturbance in CP2305 group accompanied by electroencephalogram changes, reduction in salivary chromogranin and resolution of stress-related microbiota changes	^125^
Israel	Responses to self-defence training in soldiers (*n* = 16)	Inactivated *Bacillus coagulans* 1 × 10^9^ once daily vs placebo	2 weeks	No statistically significant effect on any inflammatory, endocrine or performance responses	^127^
Spain	Adults with and without latent tuberculosis (*n* = 51)	Preparation of heat-killed *Mycobacterium manresensis* in low (10^4^) or high (10^5^) dose vs placebo	2 weeks	Increased regulatory T cell response with both doses; well tolerated	^128^
China	Patients with moderate, persistent asthma	Inhaled inactivated *Mycobacterium phlei* vs salmeterol xinafoate and fluticasone propionate powder	5 days	Symptom scores and spirometry improved to the same extent in both groups	^129^
Australia	Patients with severe COPD (*n* = 38)	Inactivated, non-typable *H. influenzae* vs placebo	Three courses, each lasting 3 days on days 0, 28 and 56 and followed for up to 20 weeks	Reduced severe exacerbations by 63% and exacerbations requiring corticosteroid therapy by 56% and hospitalization by 90%	^130^
***Bacterial lysates***					
Poland	Patients with bacterial colonization of the nose and/or throat (*n* = 150)	One 3-mg tablet of the lysate containing 1 × 10^9^ of each of: *S. aureus*, *Streptococcus mitis*, *S. pyogenes*, *S. pneumoniae*, *K. pneumoniae*, *M. catarrhalis* and *H. influenzae* (Luivac, Sankyo Pharma, Japan) vs oral personalized autovaccine capsule vs placebo daily	Two treatment periods lasting 28–30 days separated by a treatment-free interval of 28–30 days; assessed at 4 and 16 weeks after the end of treatment	The autovaccine was more effective than the lysate in reducing bacterial count of *S. pneumoniae* and β-haemolytic streptococci, whereas the lysate was more effective against *H. influenzae* colonization	^182^
Italy	Patients with COPD (*n* = 288)	Lyophilized bacterial fragments derived from *S. aureus*, *Streptococcus viridans, S. pneumoniae* (six strains), *S. pyogenes*, *K. pneumoniae*, *Klebsiella ozaenae*, *M. catarrhalis* and *H. influenzae* vs placebo	One tablet sublingually daily for 10 days followed by standard therapy alone for 20 days of standard therapy each month for 3 months followed by 3 months of standard therapy alone and then 3 months of 10 days active/placebo and 20 days standard treatment	Primary outcome (25% reduction in COPD exacerbations) not met; some secondary outcomes achieved	^39^
Italy	Patients with recurrent respiratory tract infections (*n* = 160)	Lantigen B (Bruschettini Srl.), a suspension of bacterial antigens obtained from *S. pneumoniae* type 3, *S. pyogenes* group A, *B. catarrhalis*, *S. aureus*, *H. influenzae* type B and *K. pneumoniae* in oral drops vs placebo BID	4 weeks treatment followed by 2 weeks off followed by 4 weeks on and then followed for a further 6 weeks	Significant (*P* < 0.05) reduction in the number of acute infectious episodes and use of antibiotics in the active group	^35^
Bulgaria	Patients with cancer and leukopenia following chemotherapy (*n* = 78)	DEODAN, an oral preparation, obtained from lysozyme lysates of *Lactobacillus bulgaricus* strain “I. Bogdanov patent strain Tumoronecroticance B-51” ATCC 21815 TID (no placebo)	Treated until resolution of leukopenia	Recovery of white blood count (>3,000/mm^3^) between days 3 and 5 in all patients	^183^

For evidence on the health benefits of postbiotics in adults, the Cochrane Central Register of Controlled Trials and MEDLINE databases were searched for randomized controlled trials (RCTs), cohort studies, or their meta-analyses. ATCC, American type culture collection; BID, twice a day; COPD, chronic obstructive pulmonary disease; *H. influenzae*, *Haemophilus influenzae* IBS, irritable bowel syndrome; *K. pneumoniae*, *Klebsiella pneumoniae*; *L. acidophilus*, *Lactobacillus acidophilus*; *L. gasseri*, *Lactobacillus gasseri*; *M. catarrhalis*, *Moraxella catarrhalis*; *S. aureus*, *Staphylococcus aureus*; *S. pneumoniae*, *Streptococcus pneumoniae*; *S. pyogenes*, *Streptococcus pyogenes*; TID, three times a day.

Potent examples of the power and clinical importance of substances produced by microorganisms are numerous. Perhaps the most important examples are antibiotics, the first of which, penicillin, came from the mould, *Penicillium notatum*. A truly game-changing immunosuppressant ciclosporin was derived from the fungus *Tolypocladium inflatum*. A variety of other antibacterial molecules have been isolated from gut and other microbiota, including topically applied bacteriocins such as nisin^134^ and ESL5, a bacteriocin isolated from *Enterococcus faecalis* SL-5 (ref.^135^). Topical application of these substances circumvented challenges faced by an orally administered bacteriocin in the treatment of mastitis (*n* = 8) and acne vulgaris (*n* = 70), respectively. Given the increasing concerns presented by antibiotic-resistant strains of a variety of human pathogens, the exploration of the microbiota for novel antimicrobials assumes great urgency. Such substances in a purified form fall outside the scope of postbiotics as defined herein, but they could contribute to functionality of preparations of inactivated microorganisms.

#### Clinical use

Clinical use of postbiotics has been limited by issues of delivery and formulation, but these issues are being addressed^136^ and one looks forward to the realization in the clinic of the promise that basic science has shown. One group of products of microbiota–diet interactions, SCFAs, has been subjected to clinical trials in humans with some encouraging results. Butyrate enemas have been used in clinical trials to treat ulcerative colitis (some cohort trials and some open-label studies; the number of participants in individual studies ranged from 10 to 47)^137–142^ and, to a limited extent, radiation proctosigmoiditis (RCTs; the number of participants ranged from 15 to 166)^143–146^ and visceral hypersensitivity (RCT in 11 healthy volunteers)^147^. SCFA enemas have become standard therapy for diversion colitis^148–150^. However, SCFAs used as purified substances, and not as a component of an inactivated microbial preparation, would not be considered postbiotics.

#### Genetically modified organisms

Genetically modified organisms (GMOs) are used extensively in medicine and hold considerable promise as progenitor microorganisms for postbiotics for a number of clinical scenarios ranging from inflammatory bowel disease to radiation-induced mucositis and food allergy^151–157^. Some tantalizing hints of clinical efficacy have been generated for GMOs^154,157^, but regulatory challenges, as well as the court of public opinion in some regions of the world, have hampered progress in this area. Furthermore, the clinical use of preparations of inactivated GMOs as postbiotics has — to the best of our knowledge — not yet been published, although such preparations are probably in development^158^. For feed applications in animals, some products are marketed in Europe^159^. For example, PL73 (LM) is a dried, heat-inactivated bacterial biomass used as a feed material produced from an *E. coli* K-12 strain, which was genetically modified to over-produce lysine. As mentioned earlier, we have considered vaccines, including from GMOs, outside the scope of the postbiotic definition, because they already have a dedicated term.

#### Summary

It is clear that several clinical indications could benefit from the availability of effective postbiotics, including: new antimicrobials; targeted anti-inflammatory and immunoregulatory agents; novel signalling molecules that affect gut pain, sensation, secretion and motility; and agents that enhance vaccination efficacy or modulate immune responses or that exert beneficial metabolic effects via interactions with dietary components. All could have a valuable role in clinical medicine. High-quality randomized placebo-controlled (or alternately, active agent-controlled) trials will provide the ultimate proof.

### Postbiotics in infants and children

For evidence on the health benefits of postbiotics in children, the Cochrane Central Register of Controlled Trials and MEDLINE databases were searched for RCTs or their meta-analyses that compared postbiotics with placebos or no therapy (Table 3).

**Table 3:** Examples of paediatric trials with postbiotics evaluating clinical outcomes

Country/region	Participant characteristics (*n*)	Intervention and control group	Duration of the intervention	Main conclusion	Ref.
***Fermented formula (healthy infants)***					
Italy	Age 0–4 months (*n* = 90)	Fermented formula with BB C50 and ST 065 vs breastfeeding or standard infant formula	0–4 months	A 2015 systematic review^a^ showed that fermented formula, compared with the use of standard infant formula, does not offer clear additional benefits, although some benefit on gastrointestinal symptoms cannot be excluded; no negative health effects have been documented^59^	^172^
France	0–12 months (*n* = 129)	Fermented formula with BB C50 and ST 065 *vs* standard infant formula	0–12 months		^184^
France	Age 0–4 months (*n* = 30)	Fermented formula with BB C50 and ST 065 vs standard infant formula	0–4 months		^173^
France	Age 4–6 months (*n* = 968)	Fermented formula with BB C50 and ST 065 vs standard infant formula	For 5 months		^166^
France	Age 0–3 months (*n* = 109)	Fermented formula with BB C50 and ST 065 vs standard infant formula	15 days		^185^
***Fermented formula in preterm infants***					
Italy	Preterm infants 30–35 weeks of gestational age, age 0–3 days (*n* = 58)	Preterm infant formula, heat-inactivated fermented formula with BB C50 and ST 065 vs preterm infant formula	During hospital stay; 2–5 weeks	Reduced incidence of abdominal distension in infants fed preterm fermented formula	^161^
***Management of acute gastroenteritis***					
France	Age 1–48 months (*n* = 71), acute diarrhoea	Heat-killed *L. acidophilus* LB vs placebo	4 days	A 2014 meta-analysis^a^ showed that *L. acidophilus* LB reduced duration of diarrhoea in hospitalized, but not outpatient, children compared with a placebo; the chance of a cure on day 3 was similar in both groups, but *L. acidophilus* LB increased the chance of cure on day 4 (ref.^162^)	^186^
Ecuador	10 months (*n* = 80), acute diarrhoea	Heat-killed *L. acidophilus* LB *vs* placebo	4 days		^187^
Peru	Age 3 months to 4 years (*n* = 80, acute diarrhoea (less than 3 days)	Heat-killed *Lactobacillus* LB vs placebo	4.5 days		^188^
Thailand	Age 3–24 months (*n* = 73), acute diarrhoea without severe dehydration	Lyophilized heat-killed *L. acidophilus* LB vs placebo	2 days		^189^
Finland	Age <4 years (*n* = 41), acute rotavirus diarrhoea	Heat-inactivated *L. casei* vs viable *L. casei* 10^10^ CFU	5 days	Equal clinical recovery from rotavirus diarrhoea	^163^
***Prevention of common infectious diseases***					
Italy	Age 12–48 months (*n* = 377), healthy children attending day-care or preschool at least 5 days a week	Cow’s milk + postbiotics or rice with fermented milk with heat-inactivated *L. paracasei* CBA L74 vs placebo	3 months	Reduced risk of some common infectious diseases such as gastroenteritis and respiratory tract infections (including pharyngitis, laryngitis, tracheitis) observed during the study period	^165^
Italy	Age 12–48 months (*n* = 146), healthy children, attending day-care or preschool for at least 5 days a week	Lyophilized heat-killed *L. paracasei* CBA L74 vs placebo	3 months	Reduction in some common infectious diseases, such as otitis media and pharyngitis	^168^
Pakistan	Age 6–12 months (*n* = 75), healthy infants with high risk of diarrhoea-related mortality	Micronutrient sachets with heat-inactivated *L. acidophilus* vs micronutrient sachets or placebo sachets	2 months	No statistically significant difference in diarrhoea prevalence between the micronutrient with *L. acidophilus* and placebo groups	^167^
***Atopic eczema and cow’s milk allergy***					
Finland	Mean age 5.5 months (*n* = 35), infants with atopic eczema and cow’s milk allergy	EHWF + live or heat-inactivated *L. rhamnosus* GG vs placebo	Mean 7.5 weeks	Supplementation of EHWF with viable but not heat-inactivated *L. rhamnosus* GG is a potential approach for the management of atopic eczema and cow’s milk allergy	^169^
***Allergic rhinitis***					
Taiwan	Age >5 years (*n* = 90), perennial allergic rhinitis for more than 1 year	Live or heat-killed *L. paracasei* 33 or placebo	30 days	In both intervention groups, the overall quality of life improved; heat-killed *L. paracasei* 33 was not inferior to live *L. paracasei* 33; no obvious adverse effects	^190^
***Lactose malabsorption***					
Indonesia	Age 10–12 years (*n* = 86), lactose malabsorption	Killed and live *Lactobacillus helveticus* R-52 and *L. rhamnosus* R-11	2 weeks	Decrease in breath hydrogen test in both groups	^191^

BB C50, *Bifidobacterium breve* C50; EHWF, extensively hydrolysed whey formula. *L. acidophilus*, *Lactobacillus acidophilus*; *L. casei*, *Lactobacillus casei*; *L. paracasei*, *Lactobacillus paracasei*; *L. rhamnosus* GG, *Lacticaseibacillus rhamnosus*; ST 065, *Streptococcus thermophilus* 065 ^a^Based on material presented in referenced systematic reviews.

#### Fermented formulas

Fermented formulas are those that are fermented with certain lactic acid bacteria during the production process and that do not contain substantial amounts of viable bacteria in the final product. Exact acceptable levels of live microorganisms have not been established by regulatory authorities. To the extent that the microorganisms used to ferment these formulas are characterized adequately, these products would fall under the postbiotic definition. Infant formulas serve as the sole nutrition source for infants who are not being breast fed. Thus, infant formulas are heavily regulated worldwide for their nutrient content as well as any added ingredients.

In 2007, the European Society for Paediatric Gastroenterology, Hepatology and Nutrition (ESPGHAN) Committee on Nutrition reviewed the evidence on fermented infant formulas. Based on two RCTs, the Committee concluded that the available data do not allow general conclusions to be drawn on the effects of fermented formulas in infants^160^. Updated data on fermented formulas can be found in Table 3. Overall, limited available evidence suggested that the use of fermented milk formula does not offer clear additional benefits compared with standard infant formula. At the same time, no negative health effects have been documented.

Formulas for pre-term infants are not covered by the *Codex Alimentarius*, and this issue will eventually pose a challenge to the use of fermented formulas in this age category. Data on the use of fermented formula in preterm infants are limited to one RCT, which evaluated the effect of a formula fermented by *Bifidobacterium breve* and *S. thermophilus* in a total of 58 infants (gestational age 30–35 weeks)^161^. There was a reduced incidence of abdominal distension in infants fed fermented preterm formula compared with those fed standard preterm formula, as well as statistically significantly lower faecal calprotectin levels in the former group (*P* = 0.001).

#### Management of acute gastroenteritis

A meta-analysis^162^ of four RCTs of varied methodological quality, involving 304 children aged 1–48 months, showed that heat-inactivated *Lactobacillus acidophilus* LB reduced the duration of diarrhoea in hospitalized, but not outpatient, children compared with a placebo. The chance of a cure on day 3 was similar in both groups, but *L. acidophilus* LB increased the chance of a cure on day 4 of the intervention. One trial investigated the effect of heat-inactivated *L. rhamnosus* GG compared with viable *L. rhamnosus* GG in children with acute rotavirus diarrhoea. Clinical recovery from rotavirus diarrhoea was similar in both groups^163^. A recent review covers the mechanisms as suggested by several in vitro studies^164^.

#### Prevention of common infectious diseases

Data on preventing common infectious disease are inconsistent^165–168^, However, limited results pooled from two RCTs (*n* = 537) carried out in healthy children aged 12–48 months attending day-care or preschool for at least 5 days a week suggest that heat-inactivated *Lacticaseibacillus paracasei* CBA L74 (formerly known as *Lactobacillus paracasei*) might reduce the risk of diarrhoea^165,168^, pharyngitis^165,168^, laryngitis^165,168^ and otitis media^165^. By contrast, one trial^167^ investigated the effect of micronutrients (including zinc) with or without heat-inactivated *L. acidophilus* compared with a placebo in infants aged 6–12 months at high risk of diarrhoea-related mortality (defined as at least one episode of diarrhoea in the preceding 2 weeks). The prevalence of diarrhoea was 26% in the group receiving micronutrient with *L. acidophilus*, 15% in the group receiving micronutrient and 26% in the group receiving placebo. There was no statistically significant difference between the micronutrient with *L. acidophilus* and placebo groups. The authors concluded that the addition of heat-inactivated *L. acidophilus* had a negative effect in these children.

#### Cow’s milk allergy management

Kirjavainen et al.^169^ evaluated the effects of an extensively hydrolysed whey formula (EHWF) supplemented with live or killed *L. rhamnosus* GG compared with the effects of non-supplemented EHWF in 35 infants (mean age 5.5 months) with atopic eczema and cow’s milk allergy^170,171^. The authors reported statistically significant reductions in the Scoring Atopic Dermatitis scores in the EHWF group, EHWF/viable *L. rhamnosus* GG group and the EHWF/heat-inactivated *L. rhamnosus* GG group (baseline versus end of a 1-month intervention). No adverse events in the EHWF group and the EHWF/viable *L. rhamnosus* GG group were reported. However, compared with these two groups, the administration of the EHWF/heat-inactivated *L. rhamnosus* GG resulted in a significantly higher risk of diarrhoea (*P* = 0.05).

#### Non-clinical outcomes

A number of studies evaluated additional non-clinical effects^163,172–175^. For example, the use of fermented formula was found to reduce faecal pH values. However, whether the faecal pH reduction per se is of benefit is not well established. The same applies to other stool parameters, such as faecal IgA levels and bifidobacteria levels.

#### Summary

The effects of postbiotic supplementation have been studied mainly for fermented infant formulas and bacterial lysates. Overall, there is only limited evidence to suggest that these products provide a health benefit compared with non-postbiotic-containing formulas in the paediatric setting. The safety and potential harms of postbiotic interventions remain poorly explored and understood. Further multicentre studies are necessary to determine the effects and safety of different postbiotics.

## Conclusions

This panel was conceived in response to the rise of the term ‘postbiotics’ both in the scientific literature and in relation to commercial products, as well as to the concomitant lack of clarity regarding the appropriate use of the term. The panel was interested in defining useful, science-based parameters for this emerging term. By providing a definition for the term, we hope that all stakeholders will use the term appropriately, thereby assuring a common foundation for developments in the field. If this can be achieved, it will enable scientists and intellectual property lawyers to track publications on postbiotics easily. It will provide a common understanding of the term for researchers, industry, regulators and consumers. Responsible use of the term ‘postbiotic’ on a product label will compel manufacturers to meet the minimum criteria imposed by this definition, including availability of controlled studies in the target host demonstrating a health benefit.

We have also clarified how postbiotics differ from other related substances, including probiotics, prebiotics and synbiotics. The conflation of these terms leads to confusion. Furthermore, we have called out issues that should be considered when investigating postbiotics, such as the starting material, the means of inactivation and assurance of safety. Careful control of these parameters is important for reliable and repeatable research.

## Data Availability

The PubMed search data that support the plots within this paper are available from the authors upon reasonable request.
