# Publisher Correction: Publisher Correction: The International Scientific Association of Probiotics and Prebiotics (ISAPP) consensus statement on the definition and scope of postbiotics

**DOI:** 10.1038/s41575-021-00506-5

**Published:** 2021-07-29

**Authors:** Seppo Salminen, Maria Carmen Collado, Akihito Endo, Colin Hill, Sarah Lebeer, Eamonn M. M. Quigley, Mary Ellen Sanders, Raanan Shamir, Jonathan R. Swann, Hania Szajewska, Gabriel Vinderola

**Affiliations:** 1 Functional Foods Forum, Faculty of Medicine, University of Turku, Turku, Finland; 2 Institute of Agrochemistry and Food Technology-National Research Council (IATA-CSIC), Valencia, Spain; 3 Department of Food, Aroma and Cosmetic Chemistry, Faculty of Bioindustry, Tokyo University of Agriculture, Hokkaido, Japan; 4 School of Microbiology, University College Cork, Cork, Ireland; 5 APC Microbiome Ireland, University College Cork, Cork, Ireland; 6 Department of Bioscience Engineering, University of Antwerp, Antwerp, Belgium; 7 Division of Gastroenterology and Hepatology, Lynda K and David M Underwood Center for Digestive Disorders, Houston Methodist Hospital and Weill Cornell Medical College, Houston, TX USA; 8 International Scientific Association for Probiotics and Prebiotics, Centennial, CO USA; 9 Institute of Pediatric Gastroenterology, Nutrition and Liver Diseases, Schneider Children’s Medical Center, Petach Tikva, Israel; 10 Sackler Faculty of Medicine, Tel Aviv University, Tel Aviv, Israel; 11 School of Human Development and Health, Faculty of Medicine, University of Southampton, Southampton, UK; 12 Department of Metabolism, Digestion and Reproduction, Imperial College London, London, UK; 13 Department of Paediatrics, The Medical University of Warsaw, Warsaw, Poland; 14 Instituto de Lactología Industrial (CONICET-UNL), Faculty of Chemical Engineering, National University of Litoral, Santa Fe, Argentina

Correction to: *Nature Reviews Gastroenterology & Hepatology* 10.1038/s41575-021-00481-x, published online 15 June 2021.

In the original Publisher Correction relating to Salminen et al., the copyright holder was incorrect and the Publisher Correction should have been Open Access. This issue has now been corrected.

