# Author Correction: The International Scientific Association of Probiotics and Prebiotics (ISAPP) consensus statement on the definition and scope of postbiotics

**DOI:** 10.1038/s41575-022-00628-4

**Published:** 2022-05-10

**Authors:** Seppo Salminen, Maria Carmen Collado, Akihito Endo, Colin Hill, Sarah Lebeer, Eamonn M. M. Quigley, Mary Ellen Sanders, Raanan Shamir, Jonathan R. Swann, Hania Szajewska, Gabriel Vinderola

**Affiliations:** 1 Functional Foods Forum, Faculty of Medicine, University of Turku, Turku, Finland; 2 Institute of Agrochemistry and Food Technology-National Research Council (IATA-CSIC), Valencia, Spain; 3 Department of Food, Aroma and Cosmetic Chemistry, Faculty of Bioindustry, Tokyo University of Agriculture, Hokkaido, Japan; 4 School of Microbiology, University College Cork, Cork, Ireland; 5 APC Microbiome Ireland, University College Cork, Cork, Ireland; 6 Department of Bioscience Engineering, University of Antwerp, Antwerp, Belgium; 7 Division of Gastroenterology and Hepatology, Lynda K and David M Underwood Center for Digestive Disorders, Houston Methodist Hospital and Weill Cornell Medical College, Houston, TX USA; 8 International Scientific Association for Probiotics and Prebiotics, Centennial, CO USA; 9 Institute of Pediatric Gastroenterology, Nutrition and Liver Diseases, Schneider Children’s Medical Center, Petach Tikva, Israel; 10 Sackler Faculty of Medicine, Tel Aviv University, Tel Aviv, Israel; 11 School of Human Development and Health, Faculty of Medicine, University of Southampton, Southampton, UK; 12 Department of Metabolism, Digestion and Reproduction, Imperial College London, London, UK; 13 Department of Paediatrics, The Medical University of Warsaw, Warsaw, Poland; 14 Instituto de Lactología Industrial (CONICET-UNL), Faculty of Chemical Engineering, National University of Litoral, Santa Fe, Argentina

Correction to: *Nature Reviews Gastroenterology & Hepatology* 10.1038/s41575-021-00440-6, published online 4 May 2021.

The originally published article contained an error in Table 2 in which the study on *L. gasseri* CP2305 was wrongly attributed to reference 155; it should have cited reference 15: Nishida et al. Para-psychobiotic *Lactobacillus gasseri* CP2305 ameliorates stress-related symptoms and sleep quality. *J. Appl. Microbiol.* **123**, 1561–1570 (2017). This error has now been corrected in the HTML and PDF versions of the article.

